# Association of Dietary Flavonoid Intake with Serum Cotinine Levels in the General Adult Population

**DOI:** 10.3390/nu15194126

**Published:** 2023-09-25

**Authors:** Ning Zhu, Shanhong Lin, Hang Yu, Weina Huang, Chao Cao

**Affiliations:** 1 Key Laboratory of Respiratory Disease of Ningbo, Department of Respiratory and Critical Care Medicine, The First Affiliated Hospital of Ningbo University, Ningbo 315010, China; nbyyzn@163.com (N.Z.); nbhwn722@163.com (W.H.); 2 Department of Ultrasound, The First Affiliated Hospital of Ningbo University, Ningbo 315010, China; nbyylsh@163.com

**Keywords:** dietary flavonoids, serum cotinine, smoking status, weighted quantile sum, National Health and Nutrition Examination Survey

## Abstract

Cotinine, the primary metabolite of nicotine, can be utilized as a marker for active smoking and as an indicator of exposure to secondhand smoke. However, the direct relationship between dietary flavonoid intake and serum cotinine levels remains a subject of ongoing investigation. In this study, we utilized data from the National Health and Nutrition Examination Survey (NHANES) 2007–2010 and 2017–2018 to assess the association between dietary flavonoid intake and serum cotinine levels in adults through multiple linear regression analysis. A weighted quantile sum (WQS) regression model was used to assess the association of the mixture of six dietary flavonoids with serum cotinine levels in adults, which could represent the overall effect of the mixture of six dietary flavonoids. We also conducted stratified analyses by smoke status to explore multiple linear regression associations between different flavonoid intake and serum cotinine levels. A total of 14,962 adults were included in the study. Compared to the group with the lowest dietary flavonoid intake, total flavonoid intake in the second (β = −0.29 [−0.44, −0.14]), third (β = −0.41 [−0.58, −0.24]), and highest groups (β = −0.32 [−0.49, −0.16]) was inversely related to the levels of serum cotinine after adjusting the full model. An RCS model showed that when the total dietary flavonoid intake was less than 99.61 mg/day, there was a negative linear association between dietary flavonoid intake and the serum cotinine. The WQS regression model also showed that the intake of a mixture of six dietary flavonoids was significantly negatively correlated with serum cotinine levels (β = −0.54 [−0.61, −0.46], *p* <0.01), with anthocyanins having the greatest effect (weights = 32.30%). Our findings imply a significant correlation between dietary flavonoid intake and serum cotinine levels among adults. The consumption of a combination of six dietary flavonoids was consistently linked to lower serum cotinine levels, with anthocyanins displaying the most pronounced impact.

## 1. Introduction

Flavonoids, which are bioactive compounds present in various vegetables, fruits, tea, cocoa, citrus fruits, onions, and soybeans, play a significant role in promoting health [1]. They can be classified into six main subtypes based on their molecular structure: isoflavones, flavonols, flavanols, flavanones, anthocyanins (notably, a repetition), and flavones [2]. These compounds have gained attention due to their potential to combat oxidative stress and prevent chronic diseases like neurodegenerative disorders, cardiovascular issues, and cancer [3,4].

It is widely acknowledged that tobacco smoking poses a significant risk for cardiovascular diseases, strokes, respiratory ailments, diabetes, and other chronic conditions. Cotinine, a metabolite of nicotine, serves as a marker for tobacco exposure [5]. Nicotine and its derivatives have been linked to immune suppression and inflammatory responses, leading to disturbances in organ functioning [6]. After entering the body through smoking, approximately 75–80% of nicotine is converted to cotinine in the liver [7,8]. Cotinine has a considerably longer half-life of around 16 h compared to nicotine. As a result, it is utilized to gauge nicotine intake and assess the severity of tobacco use [8]. Research involving animal models has indicated that flavonoids extracted from loquat leaves could potentially mitigate the effects of tobacco-induced chronic obstructive pulmonary disease (COPD) by inhibiting inflammation and oxidative stress [9]. Furthermore, prospective cohort studies have suggested that a high dietary intake of flavonoids is associated with a reduced risk of COPD resulting from smoking [10]. Additionally, it is essential to underscore that flavonoids represent a versatile category of bioactive compounds with far-reaching implications for human health [2,3,4]. Substantive research has firmly established their significant associations with metabolic syndrome [11,12], cardiovascular diseases [13,14], respiratory system diseases [15,16], anti-tumor activities [17,18], and neurodegenerative disorders [19,20,21]. The diverse and extensive health advantages attributed to flavonoids render them invaluable compounds with the capacity to ameliorate human health and mitigate a spectrum of chronic illnesses. However, the direct relationship between dietary flavonoid consumption and serum cotinine levels remains incompletely understood, necessitating further investigation to establish a potential connection.

Here, the primary objective of our study was to examine the potential relationship between dietary flavonoids and serum cotinine levels. Therefore, this research postulates a potential link between dietary flavonoids and serum cotinine levels. Our investigation centered on various aspects of flavonoid intake, encompassing total flavonoids as well as different subclasses, within the broader American populace. Recognizing the variations in serum cotinine levels among never smokers, former smokers, and current smokers, we further explored these relationships by stratifying our analysis into three distinct subgroups.

## 2. Methods

### 2.1. Study Population

The study population for this research comprises participants from the National Health and Nutrition Examination Survey (NHANES, https://wwwn.cdc.gov/nchs/nhanes/ (accessed on 11 May 2023)). The NHANES is a comprehensive cross-sectional survey conducted by the Centers for Disease Control and Prevention (CDC) in the United States. It employs a complex multistage probability sampling design to assess the health and nutritional status of the US population. All participants provided written informed consent and study procedures were approved by the National Center for Health Statistics Research Ethics Review Board (Protocol Number: Protocol #2005-06 and Protocol #2011-17). The details of survey administration and methods can be accessed on the NHANES website (http://www.cdc.gov/nchs/nhanes.htm (accessed on 11 May 2023)).

We collected data for 29,940 individuals from NHANES 2007–2010 and 2017–2018 survey cycles. Participants were excluded if they had missing data on dietary flavonoid intake (*n* = 3715), were pregnant (*n* = 171), lacked data on hypertension assessment (*n* = 4902), or were under the age of 20 (*n* = 5400). Ultimately, our study comprised 15,752 individuals. Refer to Figure S1 for a visual representation of our study design.

### 2.2. Dietary Flavonoid Intake Measurement

Our analysis of dietary flavonoid intake data was sourced from the USDA Food and Nutrient Database for Dietary Studies (FNDDS) [22]. This database provides information on the flavonoid content of various foods and beverages. FNDDS was primarily developed to process dietary data collected from What We Eat in America (WWEIA), a component of NHANES involving dietary recalls [22]. The United States Department of Agriculture (USDA) food codes, including versions 4.1 and 5.0, were employed to link data from WWEIA and NHANES 2007–2010 to the Flavonoid Database. A total of 7273 food codes were used to extract values for total flavonoids and six flavonoid subclasses. Data on flavonoid intake for the years 2007–2010 and 2017–2018 were gathered for our study.

The commonly consumed flavonoid subclasses in the US population are as follows: isoflavones (daidzein, Genistein, glycitein), anthocyanidins (cyanidin, delphinidin, malvidin, pelargonidin, peonidin, petunidin), flavan-3-ols ((−)-epicatechin, (−)-epicatechin 3-gallate, (−)-epigallocatechin, (−)-epigallocatechin 3-gallate, (+)-catechin, (+)-gallocatechin, theaflavin, theaflavin-3,3′-digallate, theaflavin-3′-gallate, theaflavin-3-gallate, thearubigins), flavanones (eriodictyol, hesperetin, naringenin), flavones (apigenin, luteolin), and flavonols (isorhamnetin, kaempferol, myricetin, quercetin). The mean value of two 24 h dietary intake measurements for each flavonoid was used to determine the final dietary flavonoid intake. This calculation also incorporated weights based on the stratified and multistage probability sampling design of the NHANES. The total flavonoid intake was derived from the sum of 29 individual flavonoids. To estimate total and subclass-specific flavonoid intake in food, we categorized each food or beverage into 1 of 76 mutually exclusive groups, aligned with corresponding versions of the WWEIA Food Categories.

### 2.3. Serum Cotinine Levels Assessment

The blood collection procedure followed a strict protocol outlined in the NHANES Laboratory/Medical Technologists Procedures Manual (LPM). Whole blood specimens were meticulously processed, stored (–30 °C), and subsequently shipped to the Division of Laboratory Sciences at the National Center for Environmental Health, Centers for Disease Control and Prevention for analysis.

Serum cotinine levels were assessed using isotope dilution high-performance liquid chromatography (HPLC) combined with atmospheric pressure chemical ionization tandem mass spectrometry (APCI MS/MS). This analytical approach involves spiking the serum sample with methyl-D3 cotinine as an internal standard. After an equilibration period, the sample is processed through a basified solid-phase extraction column. Cotinine is then extracted from the column using methylene chloride, and the resulting organic extract is concentrated. The concentrated residue is subsequently injected into a C18 HPLC column. Cotinine concentrations are determined by comparing the ratio of native to labeled cotinine in the sample against a standard curve. For the definition of tobacco exposure, serum cotinine levels equal to or greater than 10 ng/mL were considered indicative of tobacco exposure [23]. Furthermore, it is important to note that the laboratory procedures adhere to stringent quality assurance and quality control (QA/QC) protocols consistent with the 1988 Clinical Laboratory Improvement Act mandates. Detailed QA/QC instructions are available in the NHANES Laboratory/Medical Technologists Procedures Manual (LPM). For a comprehensive understanding of the methodology employed, please consult the NHANES website at the following URL: https://wwwn.cdc.gov/nchs/nhanes/continuousnhanes/labmethods.aspx?Cycle=2007-2008 (accessed on 11 May 2023).

### 2.4. Covariates

Numerous covariates were considered in our analysis. These variables, collected through home interviews or laboratory measurements, encompassed age, sex, race/ethnicity, education level, family poverty income ratio (PIR), drinking status, energy intake levels, body mass index (BMI), smoking status, and supplement use. Race was categorized into five groups: Mexican American, Other Hispanic, Non-Hispanic White, Non-Hispanic Black, and Other. Education level was stratified into three categories: below high school, high school, and above high school. The family PIR was segmented into three tiers: ≤1.0, 1.1–3.0, and >3.0. Drinking status was classified as nondrinker, low-to-moderate drinker (men consuming fewer than 2 drinks per day and women consuming fewer than 1 drink per day), or heavy drinker (men consuming 2 or more drinks per day and women consuming 1 or more drinks per day) [24]. The BMI was divided into 3 groups: <25.0 kg/m^2^, 25.0–29.9 kg/m^2^, and >29.9 kg/m^2^. Poverty levels were assessed using PIR, categorized into three groups as per guidelines from the US Department of Health and Human Services: ≤1.0, 1.1–3.0, or >3.0 [24]. Smoking status was classified as never smokers, former smokers, or current smokers [24].

### 2.5. Statistical Analysis

To ensure accurate calculations in the context of complex survey analysis, we incorporated a 3-cycle sample weight into our study as recommended by NHANES. Baseline characteristics were presented as percentages for categorical variables. The Chi-square test was employed to compare individuals’ characteristics between two groups based on serum cotinine levels (<10 ng/mL and >10 ng/mL) for categorical data. Spearman correlation analysis was conducted to determine correlation coefficients among all flavonoid subclasses.

To achieve a more normally distributed data, continuous dietary flavonoid intake and serum cotinine levels were transformed using the natural logarithm (ln) function. Based on levels of dietary flavonoid intake, individuals were categorized into four distinct groups. Among the six types of flavonoid intake, 57.8%, 35.2%, and 38.3% of the population reported zero intake of isoflavones, anthocyanidins, and flavanones, respectively. Consequently, individuals with no intake of isoflavones, anthocyanidins, and flavanones were placed in the first group, while those with non-zero intake were divided equally into three groups. Due to the fact that the proportion of individuals with zero intake of flavan-3-ols, flavones, and flavonols was less than 25%, the participants were evenly divided into four groups.

The association between dietary flavonoid intake and serum cotinine levels in adults was determined using multiple linear regression, accounting for different flavonoid categories. We calculated β values and corresponding 95% confidence intervals (CIs). Two models were utilized for adjustment: Model 1 encompassed age, sex, race/ethnicity, education level, family PIR, drinking status, energy intake levels, BMI, and supplement use; Model 2 included the variables from Model 1 plus smoking status. Additionally, the potential nonlinear association between the six dietary flavonoids, total flavonoids, and serum cotinine levels was explored through restricted cubic spline (RCS) analysis. We further performed stratified analyses based on smoking status to investigate multiple linear regression associations between the four flavonoids and serum cotinine levels. 

The weighted quantile sum (WQS) regression model was employed to assess the relationship between the mixture of six dietary flavonoids and serum cotinine levels in adults. This model captures the collective effect of the flavonoid mixture and calculates the contribution of each flavonoid subclass to the WQS index using corresponding weights. The weight calculation for each flavonoid intake involved bootstrap sampling (*n* = 2000), dividing the data into training (40% samples) and test (60% samples) sets. The training set determined the weights, while the test set calculated mixture significance. Further details about the WQS calculation method can be found in the literature [25]. A significance criterion was established at *p*-value < 0.05 or CIs not containing 1. All statistical analyses were performed using R software (version 4.1.0).

## 3. Results

### 3.1. Basic Characteristics of Participants

A total of 15,752 adults were enrolled in this study, and their baseline characteristics are summarized in Table 1. The study population was divided into two groups based on serum cotinine levels: those with serum cotinine levels <10 ng/mL (*n* = 8828) and those with levels ≥10 ng/mL (*n* = 6924). Individuals with serum cotinine levels ≥10 ng/mL tended to have the following characteristics: younger, male, Non-Hispanic White, higher education, middle income, current smokers, low-to-moderate drinkers, lower BMI, higher energy intake, and without supplement use. Refer to Table S1 for survey-weighted sociodemographic and health status characteristics of participants based on flavonoid intake categories.

### 3.2. Distribution and Concentration of Dietary Flavonoid Intake

The distribution and concentrations of dietary flavonoid intake among adults are presented in Table S2. The total sum of all 29 flavonoids was 208.90 mg/day, comprising 1.75 mg/day of total isoflavones, 11.77 mg/day of total anthocyanidins, 162.78 mg/day of total Flavan-3-ols, 13.60 mg/day of total flavanones, 0.87 mg/day of total flavones, and 18.13 mg/day of total flavonols. Visualized in Figure S2 are the Spearman correlation coefficients reflecting the relationships among different subclasses of dietary flavonoids. These correlations ranged from weak (r = 0.08) to strong (r = 0.67 for flavan-3-ols and flavonols).

### 3.3. The Associations of Dietary Flavonoid Intake with Serum Cotinine Levels

Table 2 presents the outcomes of multiple linear regression analyses that investigate the associations between dietary flavonoid intake and serum cotinine levels among adults. For each category of flavonoid intake, three models were employed to assess the associations, adjusting for different sets of covariates. Upon applying Model 2, which accounts for all covariates in Model 1 as well as smoking status, the results reveal distinct associations between different flavonoid categories and serum cotinine levels. Specifically, the dietary intake of flavonoids was found to be inversely associated with serum cotinine levels adjusting for Model 2. In comparison to Group 1 (reference), the following coefficients (β) and 95% CIs depict these associations: Total flavonoids: Group 2 (β = −0.29 [−0.44, −0.14]), Group 3 (β = −0.41 [−0.58, −0.24]), Group 4 (β = −0.32 [−0.49, −0.16]), with a *p*_trend_ < 0.01; Isoflavones: Group 2 (β = −0.17 [−0.31, −0.02]), Group 3 (β = −0.31 [−0.47, −0.14]), Group 4 (β = −0.37 [−0.51, −0.23]), with a *p*_trend_ < 0.01; Anthocyanidins: Group 2 (β = −0.34 [−0.52, −0.16]), Group 3 (β = −0.45 [−0.61, −0.29]), Group 4 (β = −0.55 [−0.69, −0.41]), with a *p*_trend_ < 0.01; Flavan-3-ols: Group 2 (β = −0.24 [−0.42, −0.06]), Group 3 (β = −0.34 [−0.51, −0.17]), Group 4 (β = −0.25 [−0.42, −0.08]), with a *p*_trend_ = 0.01; Flavanones: Group 2 (β = −0.29 [−0.43, −0.16]), Group 3 (β = −0.28 [−0.46, −0.10]), Group 4 (β = −0.41 [−0.58, −0.25]), with a *p*_trend_ < 0.01; Flavones: Group 2 (β = −0.34 [−0.51, −0.17]), Group 3 (β = −0.32 [−0.48, −0.17]), Group 4 (β = −0.42 [−0.58, −0.26]), with a *p*_trend_ < 0.01; Flavonols: Group 2 (β = −0.17 [−0.35, 0.00]), Group 3 (β = −0.21 [−0.34, −0.07]), Group 4 (β = −0.14 [−0.27, −0.02]), with a *p*_trend_ = 0.04. These results collectively underscore the robust and consistent negative associations between dietary flavonoid intake and serum cotinine levels.

**Figure 1:**
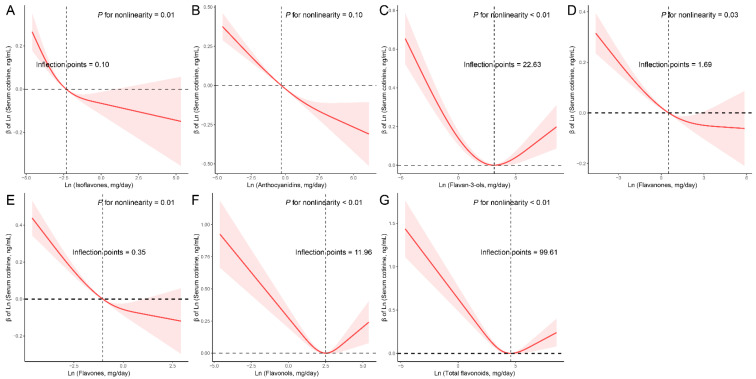
Restricted cubic spline (RCS) analysis with multivariate-adjusted associations between dietary flavonoids ((**A**) Isoflavones; (**B**) Anthocyanidins; (**C**) Flavan-3-ols; (**D**) Flavanones; (**E**) Flavones; (**F**) Flavonols; (**G**) Total flavonoids) and serum cotinine levels in adults. Models are adjusted for age (20–39, 40–59, or ≥60), sex (male or female), and race/ethnicity (Mexican American, Other Hispanic, Non-Hispanic White, Non-Hispanic Black, or Other), education level (below high school, high school, or above high school), family PIR (≤1.0, 1.1–3.0, or >3.0), drinking status (nondrinker, low-to-moderate drinker, or heavy drinker), energy intake levels (in quartiles), BMI (<25.0, 25.0–29.9, or >29.9), supplement use (yes or no), and smoking status (never smoker, former smoker, or current smoker).

Subsequently, we conducted RCS analyses to explore potential nonlinear connections between the six dietary flavonoid intake categories and serum cotinine levels (Figure 1). We identified a linear inverse relationship between anthocyanidins and serum cotinine levels (*p* for nonlinearity = 0.10). In contrast, non-linear associations were observed between total flavonoids and the other five flavonoid subclasses with serum cotinine levels. The RCS model unveiled specific threshold values where distinct linear associations emerged. For instance, when the intake of isoflavones was less than 0.10 mg/day, a linear negative correlation between dietary isoflavones intake and serum cotinine levels was evident. Similarly, dietary flavan-3-ols intake demonstrated a negative linear connection with serum cotinine levels when intake was below 22.63 mg/day. For flavanones, the linear relationship was noticeable when intake was under 1.69 mg/day, while for flavones, the threshold was 0.35 mg/day. In the case of flavonols, a linear inverse connection manifested when intake was less than 11.96 mg/day. Concerning total flavonoids, a negative linear association was observed when intake remained below 99.61 mg/day.

### 3.4. Subgroup Analysis

We further categorized the participants based on their smoking status and conducted a more refined analysis, revealing the inverse relationship between dietary flavonoid intake and serum cotinine levels within each smoking status subgroup (Table 3). Notably, there was a borderline significant interaction between anthocyanidins intake and smoking status regarding their impact on serum cotinine levels (*p* for interaction = 0.02). However, no significant interactions were observed between smoking status and serum cotinine levels across the other five flavonoid subclasses groups as well as total flavonoids. It is important to note that the results remained consistent with the aforementioned findings, maintaining the same pattern across all covariates and adjustments, aside from the variable used for stratification, which was smoking status.

### 3.5. The Associations between the Mixture of Six Dietary Flavonoid Intake and the Serum Cotinine Levels

**Figure 2:**
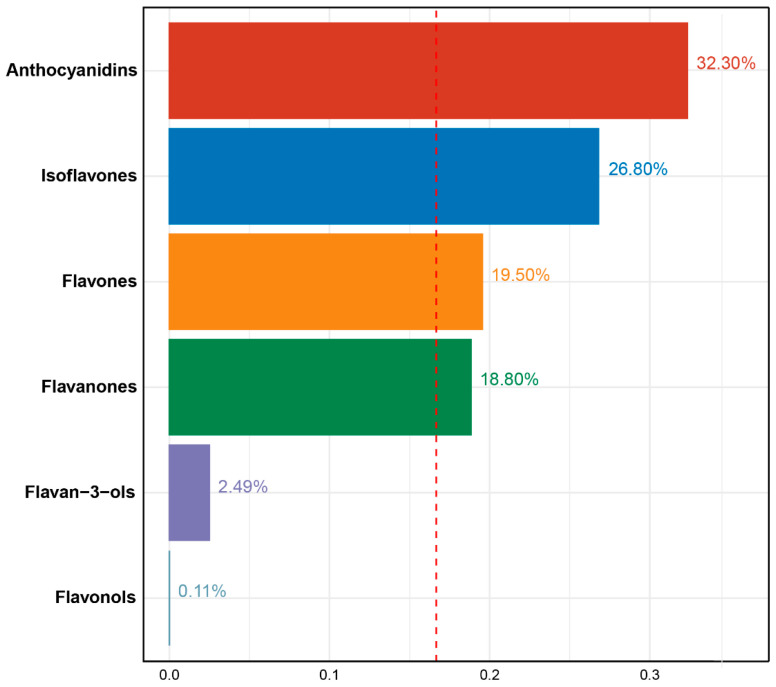
Weights from weighted quantile sum regression (WQS) for mixture of six dietary flavonoids in relation to serum cotinine levels in adults. Models are adjusted for age (20–39, 40–59, or ≥60), sex (male or female), and race/ethnicity (Mexican American, Other Hispanic, Non-Hispanic White, Non-Hispanic Black, or Other), education level (below high school, high school, or above high school), family PIR (≤1.0, 1.1–3.0, or >3.0), drinking status (nondrinker, low-to-moderate drinker, or heavy drinker), energy intake levels (in quartiles), BMI (<25.0, 25.0–29.9, or >29.9), supplement use (yes or no), and smoking status (never smoker, former smoker, or current smoker).

The WQS regression analysis showed that a significant negative association between the mixture of six dietary flavonoid intake types and serum cotinine levels (β = −0.54, 95%CI: [−0.61, −0.46], *p* < 0.01). The dietary flavonoid intake with the largest weight in the serum cotinine levels was anthocyanidins (32.30%), followed by Isoflavones (26.80%), flavones (19.50%), flavanones (18.80%), and flavan-3-ols (2.49%), and the detailed weight of each dietary flavonoid intake is presented in Figure 2.

## 4. Discussion

In this study, we observed a negative association between dietary flavonoid intake and serum cotinine levels. Subgroup analysis further revealed distinct differences among never smokers, former smokers, and current smokers, with the most pronounced differences observed in anthocyanidin consumption. Additionally, we identified that when total dietary flavonoid intake fell below 99.61 mg/day, a linear inverse relationship existed between dietary flavonoid intake and serum cotinine levels. The WQS regression model also underscored a significant negative association between the collective intake of six dietary flavonoids and serum cotinine levels, with anthocyanidins exerting the most substantial influence. This study constitutes the first attempt to estimate the connection between dietary flavonoid intake and serum cotinine levels.

While the detailed mechanisms underpinning the relationship between dietary flavonoids and serum cotinine levels remain to be fully elucidated, several plausible explanations warrant consideration. Firstly, numerous studies have indicated that flavonoids possess the capacity to regulate oxidative stress and suppress inflammatory responses. This includes the reduction in inflammatory cytokines such as nitric oxide (NO) radicals, tumor necrosis factor-alpha, interleukin-1, and interleukin-6 [26,27]. Secondly, flavonoids have been shown to modulate free radical production and the expression of transcription factors [28]. Additionally, after absorption, dietary flavonoids can undergo degradation into smaller metabolites by intestinal bacteria, potentially enhancing their bioavailability and resulting in stronger anti-androgenic effects following deglycosylation [29]. 

Nicotine, a prominent toxic component of tobacco, arises from the combustion process [30]. Cotinine, a major nicotine metabolite, is produced within the body. Previous research has demonstrated that nicotine generates a range of harmful substances, including free radicals, reactive oxygen species (ROS), and other oxidative-inducing agents [31]. Upon absorption through respiration during smoking, these toxic substances contribute to an excessive production of ROS, which can directly damage cells and tissues. Moreover, oxidative stress can impact the activity of antioxidant enzymes, weakening tissue reconstruction and cellular resistance [32]. Nicotine contributes to an imbalance in oxidation/antioxidation activity within the body. Smoking has been identified as a primary cause of numerous diseases, including cardiovascular diseases, COPD, diabetes, and cancer. The highly reactive nature of the free radicals generated by nicotine can lead to cellular and subcellular structural damage, affecting proteins, nucleic acids, and lipids [33]. Excessive oxidative damage can trigger severe inflammatory reactions, resulting in ROS accumulation [34]. Subsequently, these ROS stimulate the production of neutrophil-driven myeloperoxidase, nicotinamide-adenine dinucleotide phosphate (NADPH)-oxidases, xanthine/xanthine oxidase, and other oxidant enzymes [34,35]. Furthermore, ROS inhibit the activity of antioxidant enzymes [34,35].

Flavonoids have recently garnered attention as potential treatments for a wide array of human ailments, spanning neurodegenerative diseases, inflammation, cancer, cardiovascular health, Type 2 diabetes, and obesity, owing to their promising health benefits [3]. These bioactive compounds have demonstrated the ability to regulate pivotal cell signaling pathways, such as the arachidonic acid pathway [3]. Notably, flavonoids serve as potent anti-inflammatory and antioxidant agents. Emerging research has unveiled advantageous effects of flavonols on various neurodegenerative diseases, including Alzheimer’s disease, Parkinson’s disease, and stroke, largely attributed to their modulation of NK-κB and NLRP3 inflammasome pathways [36]. Moreover, dietary flavonoids have the capacity to traverse the blood–brain barrier, countering the detrimental effects of oxidative stress on brain cells and tissues [37]. Additionally, flavonoids may contribute to the microbiota–gut–brain axis, inhibiting toxic reactions [37]. Recent studies have demonstrated an inverse relationship between increasing dietary intake of total flavonoids and the risk of cardiovascular diseases, including ischemic heart disease [13]. Flavonoids play a crucial role in lipid metabolism, platelet aggregation, and endothelial function [27]. Anti-hypertensive effects of flavonols (especially kaempferol and quercetin) are associated with the regulation of NO by controlling NO-synthase activity and the inhibition of tyrosine kinase Pyk2 [38]. Remarkably, dietary flavonoid intake (including rutin, hesperidin, quercetin, kaempferol, and naringenin) may preclude myocardial infarction and reduce injury to myocardial ischemic cells through the inhibition of oxidative activities [38,39]. Furthermore, flavonoids (such as epigallocatechin, genistein, and resveratrol) exhibit positive effects against cancers such as breast, colon, epithelial, and prostate cancers [40]. Emerging evidence suggests that flavonoids may regulate the expression of specific tumor suppressor genes, impeding cancer progression [40]. The intake of certain flavonoids, including peonidin, naringenin, and catechin, has been inversely correlated with reduced cancer mortality [41]. In addition to their antioxidant capabilities, catechin, abundant in green tea, can modulate the cell cycle, downregulate receptor tyrosine kinase pathways, influence the immune system, and regulate epigenetic modifications [41,42]. Furthermore, prospective cohort studies have indicated that dietary intake of flavanols, flavonols, flavan-3-ols, and isoflavones could reduce the risk of Type 2 diabetes by regulating hepatic enzyme activity and glucose metabolism [43]. Flavonoids may enhance anti-diabetic effects through their regulation of hepatic enzyme activities and glucose metabolism [44]. In essence, dietary flavonoids exhibit a wide array of protective effects against various human diseases. Thus, there is a need for further exploration of potential benefits conferred by flavonoids.

Recent studies have suggested that flavonoids may hold promise in modulating the inflammation and oxidative stress triggered by tobacco smoking [10,45]. Our own findings also contribute to the growing body of evidence suggesting that flavonoids could offer protection against diseases induced by cigarette smoking. Researchers have demonstrated that diets rich in flavonoids can decrease the risk of cigarette smoking-induced COPD by attenuating smoking-induced inflammation and oxidative stress [46]. In a comprehensive cohort study spanning about 20 years, individuals who were both current smokers and had a lower total flavonoid intake faced a higher risk of cigarette-induced COPD compared to those with higher flavonoid consumption [10]. Additionally, in vitro cell-based experimental models have shown that whole-fruit consumption and intake of flavanones (a group of antioxidants abundant in fruit) increases leukocyte mitochondrial DNA copy number (mtDNAcn), whereas cigarette smoke reduces leukocyte mtDNAcn [47]. Leukocyte mtDNAcn is being explored as a potential biomarker of inflammation and oxidative responses [47]. These findings hint at a potential overlap between the mechanisms of dietary flavonoid intake and the pathways affected by cigarette smoking, particularly with respect to nicotine, a primary component of cigarettes. Furthermore, recent research has indicated an inverse association between total flavonoid intake and current smoking [48]. Cigarette smoking has been linked to increased expression of miR-21 in chronic inflammation responses, while flavonoids have shown the potential to counteract this elevation in miR-21 expression induced by cigarette smoking [49]. Additionally, studies have demonstrated that fermented black barley containing flavonoids from lactobacillus kisonensis may mitigate the harmful effects of tobacco smoking by regulating dysbiosis in gut microbiota and metabolic imbalances in lipids and amino acids [50]. Consequently, gut microbiota may wield a pivotal role in the shared pathways of dietary flavonoid intake and serum cotinine levels. These insights underscore the importance of smoking cessation and highlight the potential benefits of increasing dietary flavonoid intake. While various hypotheses have been proposed to elucidate how dietary flavonoids could counteract the effects of cigarette smoking, the specific mechanisms underlying these interactions in different contexts remain a subject of ongoing investigation. 

The strength of our research lies in its substantial sample size, which enhances the robustness and generalizability of our findings. With a large and diverse sample, our study possesses a heightened ability to capture nuanced associations between dietary flavonoid intake and serum cotinine levels across various subgroups. Additionally, our research may provide valuable insights into unraveling the connection between dietary flavonoids and diseases induced by cigarette smoking. However, several limitations must be acknowledged. Firstly, the foundation of our study on cross-sectional data impedes our ability to establish a definitive causal relationship between dietary flavonoid intake and serum cotinine levels. While our findings suggest associations, the temporal sequence and underlying mechanisms remain subject to further investigation through longitudinal studies. Secondly, our investigation primarily focused on the influence of flavonoids as constituents of dietary intake. It remains uncertain whether isolated or purified flavonoids exert similar effects, necessitating additional research to ascertain their potential health impact. Thirdly, the reliance on self-reported measures to assess flavonoid intake introduces the potential for measurement errors, which might result in inaccuracies when quantifying actual consumption. Lastly, the data exclusively encompassed adults in the United States, implying that the results might diverge when applied to other demographic groups, such as children and adolescents. Despite these limitations, it is worth noting that our study stands as the pioneering endeavor to explore the intricate relationship between dietary flavonoid intake and serum cotinine levels. As such, it contributes significantly to the current knowledge base in this area.

## 5. Conclusions

Our findings suggested that dietary flavonoid intake was associated with serum cotinine levels. The intake of a mixture of six dietary flavonoids was significantly negatively correlated with serum cotinine levels, with anthocyanins having the greatest effect. This suggests that individuals with higher dietary flavonoid intake may experience reduced exposure to environmental tobacco smoke, potentially lowering their risk of associated health problems. The significance of disseminating our findings to the scientific community lies in the potential for informed dietary recommendations and public health interventions. If future research confirms our initial observations, promoting a diet rich in flavonoids could be a cost-effective strategy for reducing exposure to tobacco-related toxins and improving public health outcomes. However, these findings need more fundamental studies and clinical trials to prove.

## Figures and Tables

**Table 1:** Survey-weighted, sociodemographic and health status characteristics of adult NHANES 2007–2010 and 2017–2018 participants with available data.

Characteristics	Total (*n* = 15,752)	Serum Cotinine, ng/mL		*p* Value
		<10 ng/mL (*n* = 8828)	≥10 ng/mL (*n* = 6924)	
Age, years				<0.01
20–39	4675 (31.25)	3175 (32.70)	1500 (45.09)	
40–59	4922 (32.9)	3524 (37.36)	1398 (39.17)	
≥60	5365 (35.86)	4497 (29.94)	868 (15.74)	
Sex, %				<0.01
Female	7591 (50.74)	6065 (54.44)	1526 (40.83)	
Male	7371 (49.26)	5131 (45.56)	2240 (59.17)	
Race/ethnicity, %				<0.01
Mexican American	2485 (16.61)	2117 (9.46)	368 (5.55)	
Other Hispanic	1539 (10.29)	1277 (5.99)	262 (4.22)	
Non-Hispanic White	6729 (44.97)	4834 (67.65)	1895 (68.95)	
Non-Hispanic Black	2931 (19.59)	1942 (9.19)	989 (14.88)	
Other race	1278 (8.54)	1026 (7.71)	252 (6.41)	
Education level, %				<0.01
Below high school	3952 (26.41)	2746 (13.98)	1206 (23.59)	
High school	3554 (23.75)	2407 (22.22)	1147 (34.32)	
Above high school	7456 (49.83)	6043 (63.80)	1413 (42.09)	
Family PIR, %				<0.01
≤1.0	3070 (20.52)	1900 (10.62)	1170 (23.39)	
1.1–3.0	6523 (43.6)	4849 (34.65)	1674 (40.34)	
>3.0	5369 (35.88)	4447 (54.74)	922 (36.27)	
Smoking status, %				<0.01
Never smoker	8100 (54.14)	7715 (69.67)	385 (10.41)	
Former smoker	3754 (25.09)	3302 (28.73)	452 (14.38)	
Current smoker	3108 (20.77)	179 (1.59)	2929 (75.21)	
Drinking status, %				<0.01
Nondrinker	3546 (23.7)	3034 (21.50)	512 (12.93)	
Low-to-moderate drinker	10,115 (67.6)	7446 (71.01)	2669 (70.49)	
Heavy drinker	1301 (8.7)	716 (7.49)	585 (16.58)	
Body mass index, %				<0.01
<25.0 kg/m^2^	4047 (27.05)	2758 (27.22)	1289 (34.66)	
25.0–29.9 kg/m^2^	5024 (33.58)	3831 (33.24)	1193 (31.57)	
>29.9 kg/m^2^	5891 (39.37)	4607 (39.54)	1284 (33.77)	
Total energy intake, kcal/day				<0.01
Quartile 1	3745 (25.03)	2915 (22.07)	830 (19.76)	
Quartile 2	3744 (25.02)	2924 (25.42)	820 (20.50)	
Quartile 3	3736 (24.97)	2832 (26.62)	904 (25.18)	
Quartile 4	3737 (24.98)	2525 (25.88)	1212 (34.56)	
Supplement use, %				<0.01
No	7477 (49.97)	5021 (41.70)	2456 (63.27)	
Yes	7485 (50.03)	6175 (58.30)	1310 (36.73)	

Abbreviations: PIR, poverty income ratio. Categorical variables are presented as numbers (percentages). Sampling weights were applied for calculation of demographic descriptive statistics; *n* reflects the study sample, while percentages reflect the survey-weighted data.

**Table 2:** Multiple linear regression associations of dietary flavonoid intake with serum cotinine levels in adults.

Flavonoids	Category of Flavonoid Intake, mg/day				*p*_trend_
	Group 1	Group 2	Group 3	Group 4	
Isoflavones					
Crude	Ref (0.00)	−0.7 (−0.95, −0.44)	−0.83 (−1.13, −0.53)	−1.02 (−1.28, −0.76)	<0.01
Model 1	Ref (0.00)	−0.45 (−0.68, −0.21)	−0.62 (−0.89, −0.35)	−0.68 (−0.91, −0.44)	<0.01
Model 2	Ref (0.00)	−0.17 (−0.31, −0.02)	−0.31 (−0.47, −0.14)	−0.37 (−0.51, −0.23)	<0.01
Anthocyanidins					
Crude	Ref (0.00)	−1.39 (−1.61, −1.17)	−1.75 (−2.03, −1.47)	−2.3 (−2.55, −2.06)	<0.01
Model 1	Ref (0.00)	−0.89 (−1.14, −0.65)	−1.14 (−1.39, −0.89)	−1.44 (−1.69, −1.20)	<0.01
Model 2	Ref (0.00)	−0.34 (−0.52, −0.16)	−0.45 (−0.61, −0.29)	−0.55 (−0.69, −0.41)	<0.01
Flavan-3-ols					
Crude	Ref (0.00)	−0.68 (−0.90, −0.47)	−1.25 (−1.52, −0.98)	−1.09 (−1.36, −0.82)	<0.01
Model 1	Ref (0.00)	−0.51 (−0.70, −0.31)	−0.92 (−1.17, −0.68)	−0.64 (−0.88, −0.41)	<0.01
Model 2	Ref (0.00)	−0.24 (−0.42, −0.06)	−0.34 (−0.51, −0.17)	−0.25 (−0.42, −0.08)	0.01
Flavanones					
Crude	Ref (0.00)	−1.11 (−1.33, −0.88)	−1.32 (−1.58, −1.06)	−1.65 (−1.92, −1.38)	<0.01
Model 1	Ref (0.00)	−0.61 (−0.79, −0.42)	−0.74 (−1.00, −0.47)	−1.03 (−1.27, −0.79)	<0.01
Model 2	Ref (0.00)	−0.29 (−0.43, −0.16)	−0.28 (−0.46, −0.10)	−0.41 (−0.58, −0.25)	<0.01
Flavones					
Crude	Ref (0.00)	−1.24 (−1.52, −0.97)	−1.46 (−1.75, −1.18)	−1.74 (−2.01, −1.47)	<0.01
Model 1	Ref (0.00)	−0.87 (−1.12, −0.62)	−0.94 (−1.20, −0.68)	−1.09 (−1.33, −0.84)	<0.01
Model 2	Ref (0.00)	−0.34 (−0.51, −0.17)	−0.32 (−0.48, −0.17)	−0.42 (−0.58, −0.26)	<0.01
Flavonols					
Crude	Ref (0.00)	−0.67 (−0.93, −0.42)	−0.63 (−0.90, −0.36)	−0.54 (−0.76, −0.33)	<0.01
Model 1	Ref (0.00)	−0.45 (−0.68, −0.23)	−0.47 (−0.67, −0.27)	−0.4 (−0.58, −0.21)	<0.01
Model 2	Ref (0.00)	−0.17 (−0.35, 0.00)	−0.21 (−0.34, −0.07)	−0.14 (−0.27, −0.02)	0.04
Total flavonoids					
Crude	Ref (0.00)	−0.98 (−1.24, −0.72)	−1.56 (−1.86, −1.26)	−1.37 (−1.62, −1.12)	<0.01
Model 1	Ref (0.00)	−0.76 (−1.00, −0.52)	−1.08 (−1.34, −0.81)	−0.87 (−1.08, −0.65)	<0.01
Model 2	Ref (0.00)	−0.29 (−0.44, −0.14)	−0.41 (−0.58, −0.24)	−0.32 (−0.49, −0.16)	<0.01

Model 1 was adjusted for age (20–39, 40–59, or ≥60), sex (male or female), and race/ethnicity (Mexican American, Other Hispanic, Non-Hispanic White, Non-Hispanic Black, or Other), education level (below high school, high school, or above high school), family poverty income ratio (≤1.0, 1.1–3.0, or >3.0), drinking status (nondrinker, low-to-moderate drinker, or heavy drinker), energy intake levels (in quartiles), BMI (<25.0, 25.0–29.9, or >29.9), and supplement use (yes or no); Model 2 was adjusted as model 1 plus smoking status (never smoker, former smoker, or current smoker).

**Table 3:** Multiple linear regression associations of dietary flavonoid intake with serum cotinine levels by smoking status in adults.

Flavonoids	Never Smoker (*n* = 8100)	Former Smoker (*n* = 3754)	Current Smoker (*n* = 3108)
Isoflavones			
Group 1	Ref (0.00)	Ref (0.00)	Ref (0.00)
Group 2	−0.08 (−0.25, 0.09)	−0.42 (−0.83, 0.00)	−0.19 (−0.49, 0.11)
Group 3	−0.30 (−0.50, −0.09)	−0.49 (−0.95, −0.04)	−0.12 (−0.31, 0.07)
Group 4	−0.27 (−0.43, −0.11)	−0.75 (−1.16, −0.33)	−0.32 (−0.53, −0.11)
*p* for trend	<0.01	<0.01	<0.01
*p* for interaction	0.37		
Anthocyanidins			
Group 1	Ref (0.00)	Ref (0.00)	Ref (0.00)
Group 2	−0.25 (−0.43, −0.06)	−0.41 (−0.88, 0.05)	−0.33 (−0.53, −0.13)
Group 3	−0.27 (−0.44, −0.10)	−0.73 (−1.16, −0.31)	−0.46 (−0.77, −0.14)
Group 4	−0.38 (−0.53, −0.23)	−0.81 (−1.13, −0.49)	−0.54 (−0.87, −0.22)
*p* for trend	<0.01	<0.01	<0.01
*p* for interaction	0.02		
Flavan-3-ols			
Group 1	Ref (0.00)	Ref (0.00)	Ref (0.00)
Group 2	−0.26 (−0.38, −0.13)	−0.12 (−0.80, 0.56)	−0.18 (−0.37, 0.02)
Group 3	−0.24 (−0.41, −0.08)	−0.4 (−0.94, 0.13)	−0.33 (−0.61, −0.04)
Group 4	−0.17 (−0.36, 0.02)	−0.31 (−0.80, 0.19)	−0.25 (−0.52, 0.01)
*p* for trend	0.2	0.11	0.03
*p* for interaction	0.65		
Flavanones			
Group 1	Ref (0.00)	Ref (0.00)	Ref (0.00)
Group 2	−0.22 (−0.38, −0.05)	−0.49 (−0.90, −0.08)	−0.19 (−0.36, −0.01)
Group 3	−0.22 (−0.41, −0.04)	−0.20 (−0.77, 0.37)	−0.42 (−0.73, −0.12)
Group 4	−0.34 (−0.48, −0.19)	−0.58 (−1.01, −0.15)	−0.16 (−0.38, 0.07)
*p* for trend	<0.01	0.05	0.01
*p* for interaction	0.17		
Flavones			
Group 1	Ref (0.00)	Ref (0.00)	Ref (0.00)
Group 2	−0.33 (−0.55, −0.11)	−0.42 (−0.88, 0.04)	−0.33 (−0.59, −0.06)
Group 3	−0.28 (−0.50, −0.06)	−0.46 (−0.84, −0.07)	−0.29 (−0.49, −0.08)
Group 4	−0.32 (−0.49, −0.14)	−0.59 (−1.00, −0.17)	−0.48 (−0.77, −0.20)
*p* for trend	<0.01	0.01	<0.01
*p* for interaction	0.38		
Flavonols			
Group 1	Ref (0.00)	Ref (0.00)	Ref (0.00)
Group 2	−0.19 (−0.32, −0.06)	−0.19 (−0.78, 0.41)	−0.14 (−0.36, 0.09)
Group 3	−0.09 (−0.31, 0.13)	−0.48 (−0.93, −0.03)	−0.13 (−0.34, 0.08)
Group 4	−0.03 (−0.21, 0.15)	−0.15 (−0.58, 0.27)	−0.36 (−0.58, −0.14)
*p* for trend	0.82	0.29	0.01
*p* for interaction	0.21		
Total flavonoids			
Group 1	Ref (0.00)	Ref (0.00)	Ref (0.00)
Group 2	−0.22 (−0.37, −0.06)	−0.43 (−0.90, 0.03)	−0.18 (−0.41, 0.05)
Group 3	−0.28 (−0.46, −0.10)	−0.58 (−1.05, −0.11)	−0.35 (−0.56, −0.13)
Group 4	−0.21 (−0.41, −0.01)	−0.52 (−0.89, −0.14)	−0.31 (−0.52, −0.09)
*p* for trend	0.07	0.01	<0.01
*p* for interaction	0.36		

Model was adjusted for age (20–39, 40–59, or ≥60), sex (male or female), and race/ethnicity (Mexican American, Other Hispanic, Non-Hispanic White, Non-Hispanic Black, or Other), education level (below high school, high school, or above high school), family PIR (≤1.0, 1.1–3.0, or >3.0), drinking status (nondrinker, low-to-moderate drinker, or heavy drinker), energy intake levels (in quartiles), BMI (<25.0, 25.0–29.9, or >29.9), and supplement use (yes or no).

## Data Availability

The data used in this study are publicly available online (https://wwwn.cdc.gov/nchs/nhanes/ (accessed on 11 May 2023)).

## Supplementary Materials

The following supporting information can be downloaded at: https://www.mdpi.com/article/10.3390/nu15194126/s1, Figure S1. Flowchart of the study participants. Figure S2. Pairwise Spearman correlation coefficients among dietary flavonoids in adults. Table S1. Survey-weighted, sociodemographic and health status characteristics of adult NHANES 2007–2010 and 2017–2018 participants with available data by category of flavonoid intakes. Table S2. Distributions and concentrations of dietary flavonoid intakes (mg/day) among adults in NHANES 2007–2010 and 2017–2018.
