# Supplementary material for: Association of Dietary Flavonoid Intake with Serum Cotinine Levels in the General Adult Population
Source: Nutrients. 2023 Sep 25;15(19):4126. doi: 10.3390/nu15194126 (PMC10574452; doi:10.3390/nu15194126)
Supplement: Supplementary file 1 [file nutrients-15-04126-s001.zip › nutrients-2596943-supplementary.pdf]

Supplementary Materials

Association of Dietary Flavonoids with Serum Cotinine Levels in the General Adult Population

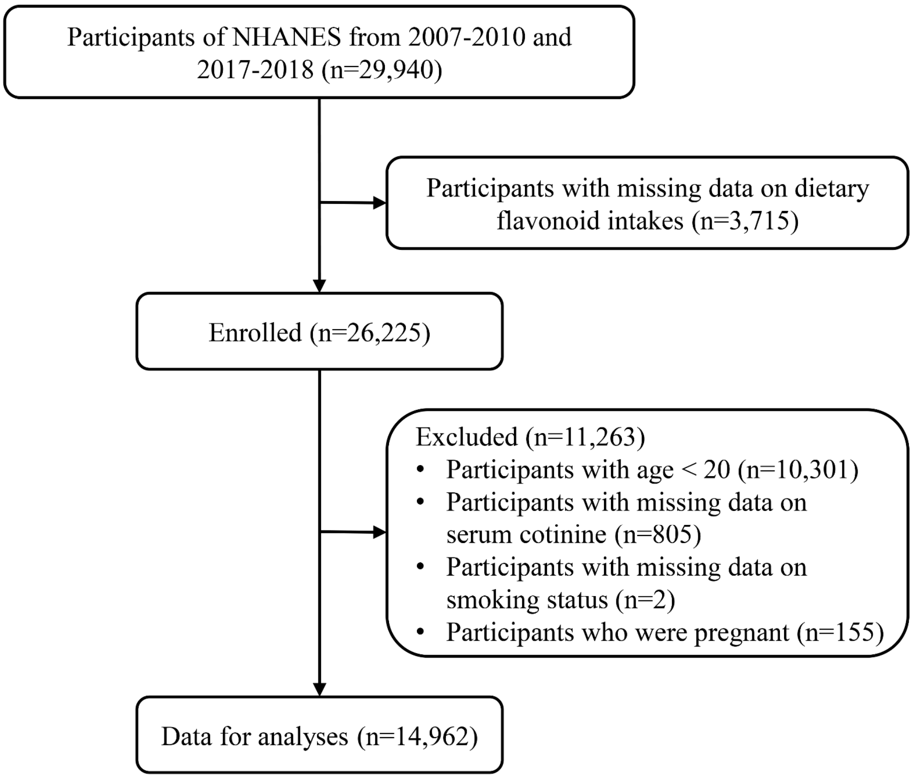

Figure S1. Flowchart of the study participants

Table S1. Survey-weighted, sociodemographic and health status characteristics of adult NHANES 2007–2010 and 2017–2018 participants with available data by category of flavonoid intakes.

Characteristics	Category of flavonoid intakes, mg/day				*P* value
	Group 1	Group 2	Group 3	Group 4	
Serum cotinine, ng/mL	0.10(0.02,143.0)	0.04(0.01,13.2)	0.03(0.01,0.49)	0.03(0.01,0.75)	<0.01
Age, years					<0.01
20-39	1319 (41.87)	1208 (36.58)	1138 (34.46)	1010 (31.06)	
40-59	1191 (35.47)	1227 (38.50)	1221 (37.22)	1283 (39.72)	
≥ 60	1228 (22.66)	1307 (24.93)	1382 (28.32)	1448 (29.22)	
Sex, %					0.48
Female	1972 (52.24)	1873 (50.04)	1856 (50.45)	1890 (51.58)	
Male	1766 (47.76)	1869 (49.96)	1885 (49.55)	1851 (48.42)	
Race/ethnicity, %					<0.01
Mexican American	622 (8.81)	754 (10.85)	698 (9.45)	411 (5.27)	
Other Hispanic	388 (5.75)	414 (6.22)	426 (6.42)	311 (4.02)	
Non-Hispanic White	1618 (65.87)	1578 (65.95)	1635 (67.46)	1898 (71.99)	
Non-Hispanic Black	868 (13.72)	746 (10.89)	664 (9.77)	653 (8.42)	
Other race	242 (5.85)	250 (6.08)	318 (6.90)	468 (10.30)	
Education level, %					<0.01
Below high school	1205 (21.42)	1075 (18.18)	910 (14.08)	762 (12.53)	
High school	1030 (31.46)	896 (24.97)	806 (22.16)	822 (22.91)	
Above high school	1503 (47.12)	1771 (56.86)	2025 (63.76)	2157 (64.56)	
Family PIR, %					<0.01
≤1.0	970 (18.84)	785 (13.90)	704 (12.04)	611 (10.94)	
1.1–3.0	1764 (41.61)	1724 (37.96)	1568 (34.27)	1467 (31.28)	
>3.0	1004 (39.55)	1233 (48.14)	1469 (53.68)	1663 (57.78)	
Smoking status, %					<0.01
Never smoker	1845 (48.67)	1978 (54.48)	2145 (57.43)	2132 (58.70)	
Former smoker	864 (23.73)	967 (25.36)	985 (27.31)	938 (24.33)	
Current smoker	1029 (27.60)	797 (20.15)	611 (15.26)	671 (16.97)	
Drinking status, %					<0.01
Nondrinker	994 (22.62)	837 (17.69)	838 (18.12)	877 (19.28)	
Low-to-moderate drinker	2508 (70.32)	2514 (70.45)	2515 (70.55)	2578 (72.03)	
Heavy drinker	236 (7.06)	391 (11.86)	388 (11.33)	286 (8.69)	
Body mass index, %					<0.01

<25.0 kg/m^2	887 (24.39)	1010 (29.31)	1079 (31.56)	1071 (30.52)	
25.0-29.9 kg/m^2	1144 (29.75)	1285 (33.13)	1291 (33.77)	1304 (34.33)	
>29.9 kg/m^2	1707 (45.86)	1447 (37.56)	1371 (34.67)	1366 (35.15)	
Total energy intakes, kcal/day					<0.01
Quartile 1	1443 (34.32)	832 (18.88)	705 (16.50)	765 (17.50)	
Quartile 2	995 (27.40)	913 (23.39)	938 (23.78)	898 (22.59)	
Quartile 3	765 (21.47)	991 (27.70)	958 (25.76)	1022 (29.53)	
Quartile 4	535 (16.81)	1006 (30.03)	1140 (33.96)	1056 (30.38)	
Supplement use, %					<0.01
No	2224 (57.36)	1930 (49.07)	1669 (40.63)	1654 (42.30)	
Yes	1514 (42.64)	1812 (50.93)	2072 (59.37)	2087 (57.70)	

Abbreviations: PIR, poverty income ratio. Categorical variables are presented as numbers (percentages). Sampling weights were applied for calculation of demographic descriptive statistics; N reflect the study sample while percentages reflect the survey-weighted data.

Table S2. Distributions and concentrations of dietary flavonoid intakes (mg/day) among adults in NHANES 2007–2010 and 2017–2018.

Flavonoid class	Mean	5th	25th	50th	75th	95th
**Isoflavones**						
Daidzein	0.68	0.00	0.00	0.00	0.01	2.46
Genistein	0.94	0.00	0.00	0.00	0.03	3.20
Glycitein	0.13	0.00	0.00	0.00	0.00	0.39
Total Isoflavones	1.75	0.00	0.00	0.00	0.04	5.74
**Anthocyanidins**						
Cyanidin	2.28	0.00	0.00	0.22	1.60	6.94
Delphinidin	1.41	0.00	0.00	0.00	0.16	5.61
Malvidin	4.15	0.00	0.00	0.00	0.15	27.93
Pelargonidin	1.41	0.00	0.00	0.00	0.13	8.95
Peonidin	1.61	0.00	0.00	0.00	0.37	4.11
Petunidin	0.92	0.00	0.00	0.00	0.05	4.16
Total anthocyanidins	11.77	0.00	0.00	0.75	6.31	61.03
**Flavan-3-ols**						
(-)-Epicatechin*	9.64	0.00	0.50	3.76	13.80	35.26
(-)-Epicatechin 3-gallate*	10.08	0.00	0.00	0.00	0.45	58.65
(-)-Epigallocatechin*	15.57	0.00	0.03	0.31	1.99	88.41
(-)-Epigallocatechin 3-gallate*	26.93	0.00	0.00	0.00	0.86	155.69
(+)-Catechin*	7.51	0.00	0.92	4.31	10.22	25.47
(+)-Gallocatechin*	1.62	0.00	0.00	0.00	0.72	9.20
Theaflavin	1.49	0.00	0.00	0.00	0.00	9.48
Theaflavin-3,3'-digallate	1.64	0.00	0.00	0.00	0.00	10.46
Theaflavin-3'-gallate	1.39	0.00	0.00	0.00	0.00	9.02
Theaflavin-3-gallate	1.18	0.00	0.00	0.00	0.00	7.46
Thearubigins	85.73	0.00	0.00	0.00	0.00	512.79
Total Flavan-3-ols	162.78	0.00	2.66	11.28	53.77	904.58
**Flavanones**						
Eriodictyol	0.22	0.00	0.00	0.00	0.03	0.98
Hesperetin	9.70	0.00	0.00	0.00	2.78	56.08
Naringenin	3.68	0.00	0.00	0.14	1.23	20.28
Total Flavanones	13.60	0.00	0.00	0.20	5.36	73.03
**Flavones**						
Apigenin	0.20	0.00	0.00	0.04	0.18	0.79
Luteolin	0.67	0.00	0.04	0.26	0.80	2.73

Total Flavones	0.87	0.00	0.08	0.38	1.07	3.21
**Flavonols**						
Isorhamnetin	0.88	0.00	0.00	0.35	1.10	3.36
Kaempferol	4.63	0.02	0.50	2.00	5.89	17.41
Myricetin	1.47	0.01	0.19	0.49	1.56	6.04
Quercetin	11.15	0.58	3.29	7.57	14.81	33.51
Total Flavonols	18.13	1.11	5.56	12.13	23.43	53.74
**Total sum of all 29 flavonoids**	208.90	3.27	16.91	48.94	172.31	989.33

5th, 5th percentile; 25th, 25th percentile; 50th, 50th percentile; 75th, 75th percentile; 95th, 95th percentile.

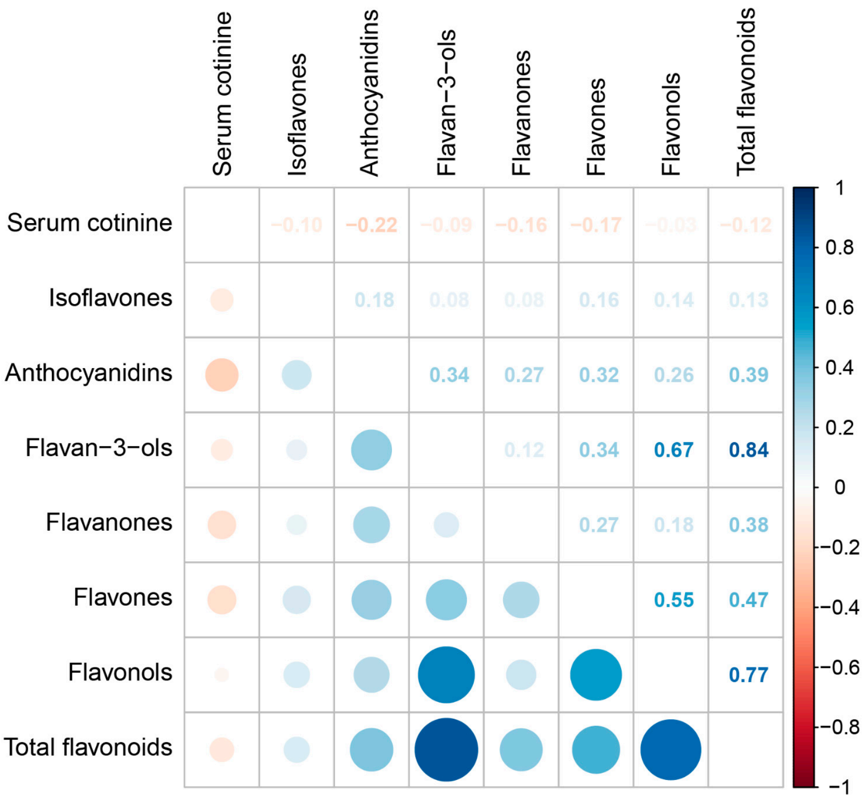

Figure S2. Pairwise Spearman correlation coefficients among dietary flavonoids in adults.
